# Evaluation of Diabetic Patients with Breast Cancer Treated with Metformin during Adjuvant Radiotherapy

**DOI:** 10.1155/2013/659723

**Published:** 2013-12-12

**Authors:** Adam Ferro, Sharad Goyal, Sinae Kim, Hao Wu, Neil K. Taunk, Devora Schiff, Aneesh Pirlamarla, Bruce G. Haffty

**Affiliations:** ^1^Department of Radiation Oncology, Rutgers Cancer Institute of New Jersey and Rutgers Robert Wood Johnson Medical School, 195 Little Albany Street, New Brunswick, New Jersey 08903, USA; ^2^Department of Biostatistics, Rutgers Cancer Institute of New Jersey and Rutgers School of Public Health, New Brunswick, New Jersey 08903, USA; ^3^Department of Radiation Oncology, Memorial Sloan Kettering Cancer Center, New York 10065, USA

## Abstract

*Purpose*. The purpose of this study was to evaluate acute locoregional toxicity in patients with breast cancer receiving concurrent metformin plus radiation therapy. *Methods and Materials*. Diabetic breast cancer patients receiving concurrent metformin and radiation therapy were matched with nondiabetic patients and diabetic patients using an alternative diabetes medication. Primary endpoints included the presence of a treatment break and development of dry or moist desquamation. *Results*. There was a statistically significant increase in treatment breaks for diabetic patients receiving concurrent metformin when compared to the nondiabetic patients (*P* value = 0.02) and a trend toward significance when compared to diabetic patients receiving an alternate diabetes medication (*P* value = 0.08). Multiple logistic regression analysis demonstrated concurrent metformin use as being associated with a trend toward the predictive value of determining the incidence of developing desquamation in diabetic patients receiving radiation therapy compared to diabetic patients receiving an alternate diabetes medication (*P* value = 0.06). *Conclusions*. Diabetic patients treated with concurrent metformin and radiation therapy developed increased acute locoregional toxicity in comparison with diabetic patients receiving an alternate diabetes medication and nondiabetic patients. Further clinical investigation should be conducted to determine the therapeutic ratio of metformin in combination with radiation therapy.

## 1. Introduction

Diabetes mellitus (DM) is a common endocrinopathy that has been shown to increase the incidence of multiple types of cancer, including breast cancer. This may stem from the upregulation of both the insulin receptor and insulin-like growth factor receptor, which promotes a dual survival and proliferation advantage through targets of the phosphatidylinositol 3-kinase/serine/threonine kinase AKT signaling pathway and activation of the mitogen kinases, MEK, and ERK, respectively [1, 2].

Metformin, a biguanide, is a first-line therapeutic agent that has been used for over 30 years in the treatment of DM. Multiple case-control analyses have demonstrated a significantly lower incidence of breast cancer in diabetic patients receiving metformin [3, 4]. The anticancer mechanism of metformin is not fully understood, but Towler and Hardie describe its involvement in the suppression of hepatic gluconeogenesis and activation of AMP-activated protein kinase (AMPK) [5]. Increased levels of AMPK reduce insulin and IGF-I signaling downstream of the receptor, therefore reducing insulin-stimulated proliferation [6]. Luo et al. describe a second mechanism involving the stimulation of LKB1, a well-recognized tumor suppressor protein with metformin intake [7]. Chlebowski et al. strengthened this assertion by observing that postmenopausal women with diabetes had a significantly lower risk of invasive breast cancer when given metformin and had a slightly higher occurrence when given a different DM drug [1]. Therefore, the anticancer effects are not solely reliant on reducing insulin-stimulated proliferation, making a second mechanism of metformin extremely relevant.

Perhaps the most exciting application for metformin will be its use as an adjuvant therapy. Breast cancer stem cells (CSCs) are resistant to conventional therapies secondary to their ability to actively export chemotherapy agents and their increased radioresistance [8]. However, Song et al. showcased metformin's aptitude as a radiosensitizer at clinically significant doses with both in vitro and in vivo murine studies, giving rise to a potential method for combating the resistant breast CSCs [9].

Metformin's novel role as a radiosensitizer may lead to a significant improvement in the prognosis of patients receiving external beam radiation therapy (EBRT) as part of breast conservation therapy or postmastectomy. However, a potential drawback of metformin is that, like other radiosensitizers, its use may increase the degree of side effects, specifically radiation dermatitis during EBRT. Radiation dermatitis is graded on a continuum, ranging from erythematous-type reactions to desquamation, skin breakdown, and ulceration. Radiation dermatitis has been shown to affect nearly 80% of patients receiving EBRT for breast cancer [10], and 31% may experience moist desquamation following standard EBRT [11]. The purpose of this study was to determine if a correlation exists between the use of metformin and radiation-induced skin toxicity in a cohort of patients treated at our institution.

## 2. Methods and Materials

After approval by the institutional review board, we used the radiation oncology database to identify patients with breast cancer who were concurrently being treated with metformin for DM during their radiotherapy; 51 patients were determined to meet this criterion. Inclusion criteria included female patients with a known diagnosis of breast cancer who were treated with concurrent metformin and radiotherapy. Male patients were excluded from the study.

Two control groups were selected by using the radiation oncology database. The first control group included nondiabetic patients (not being treated with diabetic medications). This group was matched (1 : 1) based on age (±5 years), surgical procedure, presence of adjuvant chemotherapy, radiation field design, and radiation dose. Adjuvant chemotherapy included combinations of adriamycin (A), paclitaxel (T), and cytoxan (C) (ATC, AT, or TC). A second control group included diabetic patients not treated with metformin, but treated with another diabetes medication (*n* = 28). This sample was too small to be matched in a comparable approach to the first control group. Data including demographic variables, surgery, chemotherapy, total radiation dose, radiation field design, fractionation schedule, and cancer grade and stage were extracted. Radiation treatment included either whole breast irradiation or external-beam partial-breast irradiation. Fractionation schemes for whole breast irradiation included conventional fractionation (1.8–2.0 Gy fractions to a total dose of 45–50 Gy) and hypofractionated regimens (fraction sizes >2.0 Gy). All patients that were treated with whole breast irradiation received a boost to the lumpectomy cavity or mastectomy scar.

Standard baseline evaluation included a complete medical history, physical examination, including performance status, and hematology and clinical chemistry assessments. Patients were evaluated weekly during the course of RT, 3 to 4 weeks after completion of treatment, and then at 3- to 6-month intervals thereafter. To gather information regarding locoregional toxicities, patient charts were reviewed for the development of a treatment break or desquamation (dry or moist) before, during, and after RT. A treatment break was defined as a pause in treatment, for any number of days, which was secondary to acute radiation-induced skin toxicity. In cases in which a complication could have been the result of metformin and/or radiation toxicity, it was coded as radiation toxicity unless such symptoms predated the RT.

Statistical analysis was performed using a chi-square or Fisher's exact test when appropriate, with a *P* value of 0.05 or less indicating significance. The computer program software R (version 2.15.1) was used for all statistical testing.

## 3. Results

### 3.1. Patient Characteristics

Clinical, pathologic, and treatment characteristics of the patients in each treatment group are displayed in Table 1. Patients in this study were treated with either breast-conserving surgery or mastectomy followed by radiation therapy with varying radiation field designs and dosages. Patients in the study initiated radiotherapy from November/2004 to June/2012. Patient matching was used to ensure subgroup homogeneity between patients with DM receiving metformin and the subgroup of nondiabetics (not being treated with metformin) during radiation therapy. Matching was unachievable for the subgroup of DM patients receiving a diabetes medication other than metformin because of a small sample size. The mean age of the patients was 60 years (ranging, 27 to 83). The subgroups were homogenous with respect to age, race, breast (left or right), smoking status, presence of collagen vascular disease, tumor grade, pathologic T stage, estrogen receptor positivity, progesterone receptor positivity, radiation field design, fractionation schedule, and dose max (>110% prescription dose). Only the percentage of patients receiving axillary dissection (*P* value = 0.045), separation (>25 cm) (*P* value = 0.007), and percentage of patients with Her2 receptor positivity (*P* value = 0.050) had a significant difference among subgroups.

## 4. Toxicities

### 4.1. Treatment Breaks

The incidence of a treatment break secondary to skin toxicity was determined for each patient in the study. The group of patients receiving metformin for their DM treatment had nine (18%) treatment breaks secondary to high-grade radiation dermatitis reactions. The diabetic patients not receiving metformin and the nondiabetic patients each had only one treatment break (4% and 2%, resp.). There were a statistically significant increase in the frequency of treatment breaks for diabetic patients receiving metformin compared to the nondiabetic breast cancer patients (*P* value = 0.02) and a trend toward significance when compared to diabetic patients concurrently receiving an alternate diabetes medication (*P* value = 0.08).  Table 2 displays the univariate analysis using Fisher's exact test to assess possible confounding variables amongst the three patient groups. Race was the only other predictor of developing a treatment break (*P* value = 0.012). Tumor grade was marginally significant (*P* value = 0.08).

### 4.2. Desquamation

**Figure 1:**
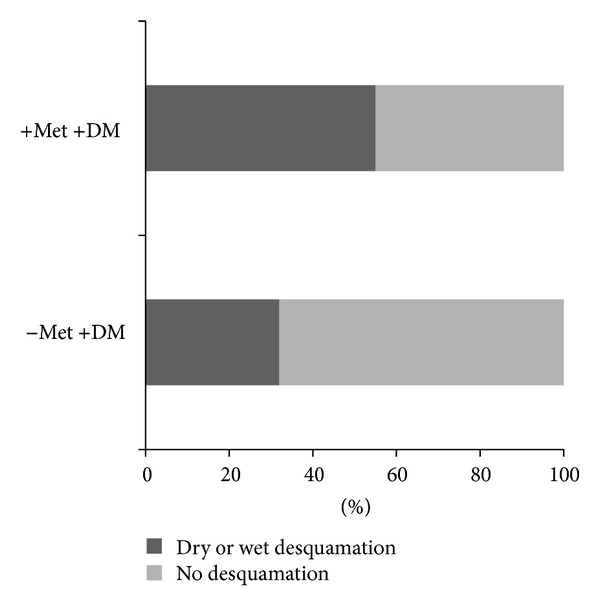
Desquamation reactions. Met: metformin, RT: radiation therapy. Acute skin toxicity comparing frequency of desquamation in patients receiving concurrent metformin and radiation therapy versus patients receiving an alternate diabetes medication and RT.

Radiation dermatitis grades categorized according to presence of desquamation are displayed in Figure 1. On univariate analysis, there was a trend toward a statistically significant increase in the frequency of desquamation reactions (dry or moist) for diabetic patients treated with metformin compared to diabetic patients being treated with a diabetes medication other than metformin (*P* value = 0.09). Twenty-eight (55%) diabetic patients treated with metformin developed desquamation. Diabetic patients using a medication other than metformin and nondiabetic patients developed desquamation 32% and 49%, respectively. The odds ratio for developing desquamation for a diabetic patient receiving concurrent metformin and EBRT is 2.57 (95% confidence interval, 0.98–6.75) when compared to diabetic patients receiving EBRT while taking another diabetes medication.  Table 3 represents a univariate analysis to assess possible confounding variables for the development of desquamation amongst each group. Radiation field design was the only significant variable (*P* value = 0.03). As expected, a larger treatment volume was predictive of developing desquamation.

### 4.3. Multivariate Analysis

Multivariate logistic regression models were performed to find predictors of desquamation reactions in diabetic patients. Initial models included age (greater than 50), surgery, left or right breast, smoking status, collagen vascular disease, axillary dissection, tumor grade, pathologic T stage, estrogen receptor status, progesterone receptor status, Her2neu receptor status, and adjuvant hormone therapy treatment. Variables were analyzed if they displayed a trend toward significance on univariate analysis (*P* value < 0.10). For the development of desquamation, radiation field design and concurrent metformin use were included in the final model. Our results demonstrated that the only field design (*P* value = 0.013) was significant in this model, while concurrent metformin demonstrated a trend toward significance (*P* value = 0.06).

## 5. Discussion

In this study, we demonstrated there to be a correlation between metformin use and an increase in locoregional toxicity in diabetic breast cancer patients receiving EBRT.

Metformin's novel role as an anticancer agent has stimulated a significant amount of research with regard to using metformin as an adjuvant therapy. Previous in vitro studies have documented metformin's aptitude as a radiosensitizing agent in human hepatic, lung prostate, and breast cancer cells [12, 13]. Although the mechanisms are not fully understood, ionizing radiation and metformin both have been demonstrated to cause a decrease in cellular survival via the activation of AMPK. Furthermore, Sanli et al. demonstrated that increased AMPK levels caused cellular arrest at the G2/M phase [13]. Arrest at this phase of the cell cycle has been shown to cause increased radiosensitivity [14].

There have been limited clinical studies addressing the role of metformin as a radiosensitizing agent. A retrospective analysis from MD Anderson demonstrated that metformin use was associated with a dose-dependent increased response to neoadjuvant chemoradiation and a decreased rate of field locoregional failure in patients with esophageal adenocarcinoma [15]. Additionally, Spratt et al. retrospectively evaluated metformin as a radiosensitizer in prostate cancer and found there to be a survival benefit (improved prostate-specific antigen-recurrence-free survival, distant metastases-free survival, prostate cancer-specific mortality, and overall survival) and reduced development of castration-resistant prostate cancer in patients treated with EBRT while concurrently receiving metformin [16]. To our knowledge, there has been no clinical literature evaluating metformin's ability to act as a radiosensitizing agent in breast cancer. Additionally, no studies have addressed toxicity profiles in diabetic breast cancer patients receiving concurrent metformin and EBRT.

In our study, we established that diabetic breast cancer patients receiving concurrent EBRT and metformin developed a statistically significant increase in the frequency of treatment breaks when compared to nondiabetic patients and a trend toward significance when compared to diabetic patients receiving an alternate DM medication. We also demonstrated that diabetic breast cancer patients receiving metformin developed a trend toward a statistically significant increase in the frequency of desquamation reactions when compared to diabetic patients receiving a diabetes medication other than metformin.

Some limitations that were identified in our study were that our ideal control group only contained twenty-eight patients and the group was not matched to the experimental group based on age (±5 years), surgical procedure, presence of neoadjuvant chemotherapy, radiation field design, and radiation dose as the nondiabetic control group was matched. Despite this, there was clearly a significant increase in the frequency of treatment breaks from that expected in the patients taking metformin concurrently with EBRT. Additionally, breast separation was heterogeneous between groups; however, breast separation was not determined to be a predictor of developing either a treatment break or desquamation, and the cohort groups were similar with respect to maximum dose. Maximum dose is a more accurate predictor of desquamation reactions than breast separation. Breast separation was used as a substitute for body mass index which has been shown to be a prognostic factor for radiation dermatitis [17]. Another recognized limitation of the study was that some of the patients receiving metformin were also being treated with a second diabetes medication. Ideally, this group should only be receiving metformin, but many patients need multiple diabetes medications to control their disease. Hardie demonstrated that thiazolidinediones and metformin activate AMPK [18]. It is possible that the thiazolidinediones may be adding to the effect of metformin to increase skin toxicity.

Ideally, a multivariate logistic regression (LR) analysis would be included as part of the statistical methods in our evaluation to ensure that concurrent metformin use is an independent risk factor for developing a treatment break. Our decision to exclude a multivariate LR analysis for the presence of a treatment break was based on the small number of patients that developed this outcome. Simulation experiments have suggested that the maximum events per predictor ratio should be at least 5 to 10 [19, 20]. Thus, in our case the maximum number of predictors in our multivariate model would only be one to two. However, it is important to note that the cohort of diabetic patients being treated with metformin was matched to the nondiabetic patient cohort with respect to age (±5 years), surgical procedure, presence of neoadjuvant chemotherapy, radiation field design, and radiation dose. Furthermore, a univariate analysis of patient, disease, and treatment characteristics demonstrated that race was the only statistically significant risk factor, other than concurrent metformin treatment, that was associated with the development of a treatment break. Treatment breaks were predominantly experienced in non-Caucasian or non-African American patients. While studies have described African American race to be a risk factor for increased acute radiation skin toxicity, to our knowledge, other races have not been identified as a risk factor [21].

Our findings are clinically significant to other clinical tumor sites because higher-grade radiation dermatitis reactions cause inferior cosmetic outcomes. Furthermore, an increase in the frequency of treatment breaks can cause an increase in the rate of locoregional failure secondary to accelerated repopulation (rapid multiplication of surviving clonogens). It has been established that extending overall treatment time is detrimental to tumor control secondary to accelerated repopulation in multiple malignancies, including squamous cell carcinomas of the pharynx and larynx, as well as cervical and bladder cancers [22]. In addition, increased accelerated repopulation has been demonstrated under hypoxic conditions [23]. Thus, diabetics may be a population of patients that have a considerably increased risk of locoregional failure. Our study demonstrates that metformin use is associated with an increase in the frequency of treatment breaks (longer overall treatment) and diabetic patients may be secondary to a damaged microvascular system causing relative hypoxia.

In our study, although we found there to be a correlation between increased acute skin toxicity and metformin use, there were no locoregional recurrences in the diabetic patients treated with metformin and two recurrences in the nondiabetic patients (no figure reported). Thus, whether concurrent metformin use with EBRT translates clinically into an improved therapeutic ratio in breast cancer remains to be seen. While our sample size is too small to draw a conclusion about metformin's potential as an adjuvant therapy, our results combined with previous metformin radiosensitization in vitro studies and the clinical studies for esophageal and prostate cancer warrant further investigation for the future role of metformin during radiation therapy.

## 6. Summary

Derived from preclinical studies demonstrating metformin-induced radiosensitization, we hypothesized that patients receiving concurrent metformin and radiation would experience increased locoregional toxicity. Our study demonstrates that patients receiving concurrent metformin and radiotherapy experience an increased frequency of treatment breaks and desquamation. Further clinical investigation should be conducted to determine the potential risks and benefits of metformin in combination with radiation therapy.

## Figures and Tables

**Table 1:** Patient characteristics.

Characteristic	Diabetics treated w/metformin	Diabetics treated with other drugs	Nondiabetics treated w/metformin	*P* value
No.	51	28	51	
Mean age	60.02	61.89	57.75	
Age > 50 years no. (%)				0.457
Yes	44 (86)	23 (82)	39 (76)	
No	7 (14)	5 (18)	12 (24)	
Race, no. (%)				0.058
White	20 (39)	12 (43)	34 (67)	
Black	9 (18)	7 (25)	6 (12)	
Other	22 (43)	9 (32)	11 (21)	
Breast no. (%)				0.103
Right	25 (49)	18 (64)	20 (39)
Left	26 (51)	10 (36)	31 (61)
Smoker no. (%)				0.462
No	34 (67)	21 (75)	42 (82)	
Previous	14 (27)	6 (21)	8 (16)	
During RT*	3 (6)	1 (4)	1 (2)	
CVD^†^ no. (%)				0.349
Yes	2 (4)	0 (0)	0 (0)	
No	49 (96)	28 (100)	51 (100)	
Axillary dissection no. (%)				**0.045**
Yes	22 (39)	8 (29)	21 (41)	
No	29 (61)	20 (71)	30 (59)	
Grade no. (%)				0.404
Low	7 (14)	4 (14)	3 (6)	
Intermediate	12 (24)	10 (36)	17 (33)	
High	21 (41)	8 (29)	16 (31)	
Unknown	11 (21)	6 (21)	15 (29)	
T stage no. (%)				0.103
In situ	6 (12)	1 (4)	0 (0)	
1	22 (43)	13 (46)	31 (61)	
2	15 (29)	8 (29)	10 (19)	
3	1 (2)	2 (7)	3 (6)	
4	1 (2)	2 (7)	3 (6)	
Unknown	6 (12)	2 (7)	4 (8)	
ER^††^ no. (%)				0.757
Positive	33 (65)	22 (79)	37 (73)	
Negative	14 (27)	6 (21)	13 (25)	
Unknown	4 (8)	0 (0)	1 (2)	
PR^§^ no. (%)				1
Positive	31 (61)	19 (68)	34 (67)	
Negative	16 (31)	9 (32)	16 (31)	
Unknown	4 (8)	0 (0)	1 (2)	
Her2^||^ no. (%)				**0.050**
Yes	6 (12)	2 (7)	15 (29)	
No	45 (88)	26 (93)	36 (71)	
Separation (>25 cm) (%)				**0.007**
Yes	28 (55)	9 (32)	13 (25)	
No	23 (45)	17 (68)	38 (75)	
Radiation Field Design				0.98
2-field	31 (61)	16 (57)	31 (61)	
SC	12 (23)	7 (25)	12 (23)	
SC + IM	3 (6)	1 (4)	3 (6)	
APBI	5 (9)	4 (14)	5 (9)	
Fractionation schedule				0.17
Conventional	40 (78)	16 (57)	41 (80)	
Hypofractionated	6 (12)	7 (25)	4 (8)	
APBI	5 (10)	5 (18)	6 (12)	
Dose max (>110%)				0.92
Yes	19 (37)	9 (32)	18 (35)	
No	32 (63)	19 (68)	33 (65)	

*Radiation therapy, ^†^collagen vascular disease, ^††^estrogen receptor, ^§^progesterone receptor, ^||^human epidermal growth factor receptor,

SC: supraclavicular nodes, IM: internal nammary nodes, APBI: accelerated partial breast irradiation.

CVD: collagen vascular disease, ER: estrogen receptor, and PR: progesterone receptor. Patients characteristics table using Fisher's exact test to compare groups.

The bold numbers refer to numbers that are statistically significant (<.05) for the variable on the same line (listed on the left).

**Table 2:** Treatment break univariate analysis.

Predictor	*P* value
Age > 50 years	1.0
Surgery	0.88
Radiation field	0.92
Race	**0.01**
Breast	0.36
Smoker	0.81
CVD	1.0
Axillary dissection	0.12
Grade	0.08
T stage	0.34
ER^§^	0.13
PR	0.29
Her2^¶^	0.68
Adjuvant hormone therapy	0.06
Dose max (>110%)	0.52
Separation (>25 cm)	0.33

^§^Estrogen receptor, ^¶^human epidermal growth factor receptor-2.

CVD: collagen vascular disease, ER: estrogen receptor, and PR: progesterone receptor. Univariate analysis to evaluate possible confounding variables using Fisher's exact test.

The bold numbers refer to numbers that are statistically significant (<.05) for the variable on the same line (listed on the left).

**Table 3:** Radiation dermatitis univariate analysis.

Predictor	*P* value
Age > 50 years	0.17
Surgery	0.48
Radiation field	**0.03**
Race	0.71
Breast	0.213
Smoker	0.84
CVD	1
Axillary dissection	1
Grade	0.079
T stage	1
ER^§^	1
PR	0.488
Her2^¶^	0.1
Adjuvant hormone therapy	1
Dose max (>110%)	0.11
Separation (>25 cm)	0.19

^§^Estrogen receptor, ^¶^human epidermal growth factor receptor-2.

CVD: collagen vascular disease, ER: estrogen receptor, and PR: progesterone receptor. Univariate analysis to evaluate possible confounding variables using Fisher's exact test.

The bold numbers refer to numbers that are statistically significant (<.05) for the variable on the same line (listed on the left).
